# Effects of Marine and Freshwater Macroalgae on *In Vitro* Total Gas and Methane Production

**DOI:** 10.1371/journal.pone.0085289

**Published:** 2014-01-22

**Authors:** Lorenna Machado, Marie Magnusson, Nicholas A. Paul, Rocky de Nys, Nigel Tomkins

**Affiliations:** 1 School of Marine and Tropical Biology, James Cook University, Townsville, Queensland, Australia; 2 Centre for Sustainable Tropical Fisheries and Aquaculture, James Cook University, Townsville, Queensland, Australia; 3 CSIRO Animal Food and Health Sciences, James Cook University, Townsville, Queensland, Australia; Mount Allison University, Canada

## Abstract

This study aimed to evaluate the effects of twenty species of tropical macroalgae on *in vitro* fermentation parameters, total gas production (TGP) and methane (CH_4_) production when incubated in rumen fluid from cattle fed a low quality roughage diet. Primary biochemical parameters of macroalgae were characterized and included proximate, elemental, and fatty acid (FAME) analysis. Macroalgae and the control, decorticated cottonseed meal (DCS), were incubated *in vitro* for 72 h, where gas production was continuously monitored. Post-fermentation parameters, including CH_4_ production, pH, ammonia, apparent organic matter degradability (OMd), and volatile fatty acid (VFA) concentrations were measured. All species of macroalgae had lower TGP and CH_4_ production than DCS. *Dictyota* and *Asparagopsis* had the strongest effects, inhibiting TGP by 53.2% and 61.8%, and CH_4_ production by 92.2% and 98.9% after 72 h, respectively. Both species also resulted in the lowest total VFA concentration, and the highest molar concentration of propionate among all species analysed, indicating that anaerobic fermentation was affected. Overall, there were no strong relationships between TGP or CH_4_ production and the >70 biochemical parameters analysed. However, zinc concentrations >0.10 g.kg^−1^ may potentially interact with other biochemical components to influence TGP and CH_4_ production. The lack of relationship between the primary biochemistry of species and gas parameters suggests that significant decreases in TGP and CH_4_ production are associated with secondary metabolites produced by effective macroalgae. The most effective species, *Asparagopsis*, offers the most promising alternative for mitigation of enteric CH_4_ emissions.

## Introduction

Methane (CH_4_) is a greenhouse gas (GHG) produced primarily by methanogenic microbes that are found in natural ecosystems (e.g. wetlands, oceans and lakes) and the gastrointestinal tract of invertebrates and vertebrates, such as termites and ruminants [1]. Every year ∼429–507 Tg of CH_4_ are removed from the atmosphere and ∼40 Tg from the stratosphere through reactions with hydroxyl (OH) radicals; and ∼30 Tg by CH_4_-oxidizing bacteria in soil [2]. Nevertheless, anthropogenic GHG emissions have been increasing rapidly, with the CH_4_ concentration in the atmosphere now more than twofold higher than in the early 1800s [3]. Methane is very effective in absorbing solar infrared radiation and has a global warming potential 25 times greater than CO_2_ [1]. Consequently, its accumulation in the atmosphere contributes considerably to climate change. One of the main sources of anthropogenic CH_4_ can be attributed to agricultural activities, particularly from ruminant livestock which are responsible for 25% of the total methane emissions in the atmosphere [2]. In Australia, ruminants are estimated to contribute ∼10% of the total GHG emissions [4], [5].

Ruminants produce CH_4_ as a by-product of the anaerobic microbial fermentation of feeds in the rumen and, to a lesser extent, in the large intestine [6]. The ruminal microbial community is highly diverse and composed of bacteria, protozoa, fungi, and bacteriophages that act collectively to ferment ingested organic matter (OM), resulting in CO_2_, H_2_, volatile fatty acids (VFAs), and formates [7]. Methanogenic archaea present in the rumen use these end-products and produce CH_4_. Although the production of CH_4_ reduces the partial pressure of H_2_, which could otherwise inhibit rumen fermentation, it also reduces the amount of energy and carbon available for formation of VFAs essential for ruminant nutrition [7], [8]. Most of the CH_4_ produced in ruminants is exhaled and belched by the animal and represents a loss of up to 12% of gross energy intake [9]. Therefore, it is essential to develop mitigation strategies that reduce enteric CH_4_ formation and result in improved feed utilization, diet digestibility, and ultimately livestock productivity [10]. By improving diet digestibility and energy use efficiency in ruminants the overall productivity may be increased and the implementation of mitigation strategies could become economically viable.

Nutritional management offers an efficient short-term strategy to reduce enteric CH_4_ emissions. Increasing the amount of grain and leguminous forages, and the use of diet supplements such as proteins, fats and oils can inhibit methanogenesis, and consequently, CH_4_ production [6], [11], [12], [13]. However, many of these grains and supplements, such as soybeans, wheat and corn, are also human food sources. The use of dietary additives, such as monensin, has been reported to reduce enteric CH_4_ production, although the effect is transient [13], [14]. Phenolic compounds, tannins and saponins are also used for this purpose [15]. Nonetheless, anti-methanogenic effects of these compounds vary according to their molecular structure, with some compounds also leading to a simultaneous decrease in feed digestibility [16].

Macroalgae are economically important providing biomass for human foods, phycocolloids and animal feed [17], [18]. They are rich in primary metabolites essential to metabolic function as minerals, vitamins, proteins, lipids and polysaccharides that can be used to improve basal feed quality [18], [19], [20], [21]. The use of macroalgae in livestock feeds can increase growth rates and feed conversion efficiency in ruminants [19] and reduce enteric CH_4_ production [22], [23]. Some species of macroalgae also produce secondary metabolites with anti-bacterial, anti-viral, antioxidant, and anti-inflammatory properties that enhance animal health and function [24], [25], but can also impair fiber degradation [22] limiting diet digestibility and animal productivity. Therefore, information about the primary biochemical profile of species of macroalgae on ruminal fermentation is crucial prior to implementation as a dietary supplement [26]. In this study we evaluated the effects of marine and freshwater species of macroalgae on fermentation parameters, total gas production (TGP) and CH_4_ production *in vitro*. Twenty species of tropical macroalgae were included providing an extensive quantitative and qualitative assessment of the use of macroalgal biomass as a natural alternative for mitigation of ruminant GHG emissions by ruminant livestock.

## Materials and Methods

### Collection and preparation of algae samples

**Figure 1:**
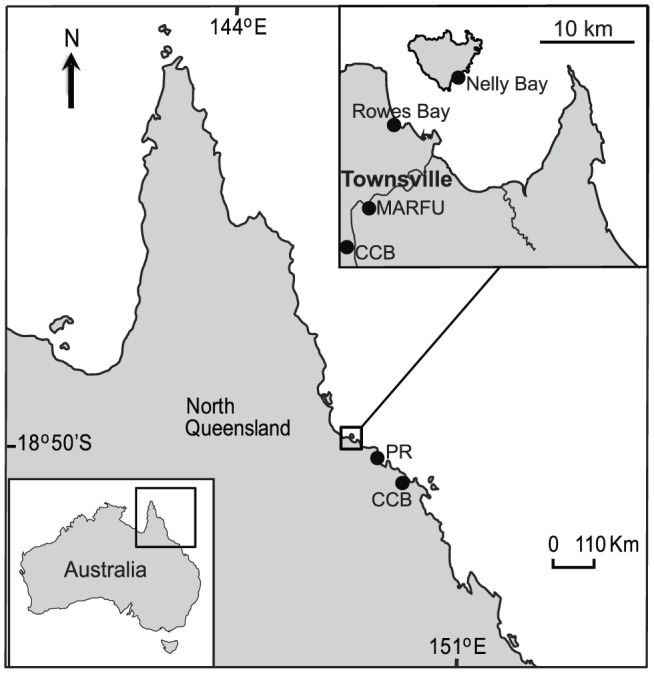
Geographic location of sampling sites included along the North Queensland's coast, Australia. Sites are represented by the dot points. MARFU: Marine and Aquaculture Research Facility Unit, Macroalgal Biofuels and Bioproducts Research Group, James Cook University (19.33°S; 146.76°E); CCB: Coral Coast Barramundi Fisheries, a barramundi farm (19.36°S; 146.70°E, Townsville, and 20.02°S; 148.22°E, Bowen); PR: Pacific Reef Fisheries, Tiger prawn farm (19.58°S, 147.40°E); Nelly Bay, an intertidal reef flat situated in Magnetic Island (19.16°S; 146.85°E), Rowes Bay, an intertidal reef flat situated in Townsville (19.23°S, 146.79°E).

Twenty species of marine and freshwater macroalgae were selected for this study based on their occurrence and abundance in aquaculture systems and intertidal areas around Townsville, Queensland, Australia (Fig. 1, Table S1). Seven species of macroalgae were harvested from large scale cultures at James Cook University (JCU), Townsville. The remaining species were collected at two intertidal reef flats: Nelly Bay, Magnetic Island (19°16′S; 146°85′E) under GBRMPA permit number GO2/20234.1; Rowes Bay (19°23′S, 146°79′E, Townsville) under DPIF permit number 103256; and from marine and freshwater aquaculture facilities in Townsville and surrounds.

All macroalgae were rinsed in freshwater to remove sand, debris and epiphytes. Biomass was centrifuged (MW512; Fisher & Paykel) at 1000 rpm for 5 min to remove excess water and weighed. A sub-sample of each species was preserved in 4% formalin for taxonomic identification, while the remaining biomass was freeze-dried at −55°C and 120 µbar (VirTis K benchtop freeze-drier) for at least 48 h. Freeze-dried samples were ground in an analytical mill through 1 mm sieve, and stored in airtight containers at −20°C until incubation.

### Biochemical parameters of substrates

The proximate and elemental composition (from here on referred to as biochemical parameters) of macroalgae, decorticated cottonseed meal (DCS) and Flinders grass (*Iseilema* sp.) hay were evaluated in duplicate (Table S1 and Table S2). Moisture content was determined using a digital moisture analyzer (A&D, MS-70, Tokyo, Japan), where 2 g samples were heated at 105°C to constant weight. The dry matter (DM) content was determined by deducting the moisture content from the total weight of the samples. Organic matter content (OM) was determined by combustion of the 2 g samples in a muffle furnace for 6 h at 550°C. Carbon, hydrogen, oxygen, nitrogen, phosphorous, and sulfur (CHONS) were quantified by elemental analysis (OEA laboratory Ltd., UK). Crude protein (CP) fraction was estimated using total nitrogen content (wt %) of the biomass with nitrogen factors of 5.13, 5.38, and 4.59 for green, brown and red macroalgae, respectively [27], and 6.25 for DCS and Flinders grass hay. Total lipid content was extracted and quantified using the Folch method [28]. Fatty acids were extracted by a one-step extraction/transesterification method and quantified as fatty acid methyl esters (FAME) by gas GC/MS/FID (Agilent 7890 GC with FID – Agilent 5975C EI/TurboMS), as described in ([29], Table S3). Carbohydrate content was determined by difference according to equation (1).
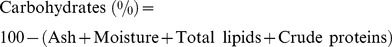
(1)Where ash, moisture, total lipids and crude proteins are expressed as a percentage of DM.

The gross energy content (GE) of each sample was calculated according to Channiwala and Parikh [30], based on elemental composition:
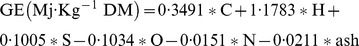
(2)Since macroalgae accumulate essential mineral elements [18] and heavy metals [31] which can inhibit anaerobic digestion [32], the concentrations of 21 elements were also quantified on 100 mg samples using ICP-MS analysis [33].

### *In vitro* experimental design

Rumen fluid was collected from three rumen fistulated *Bos indicus* steers (632±32.62 kg live weight) which were maintained at the School of Biomedical and Veterinary Sciences, JCU, according to experimental guidelines approved by CSIRO Animal Ethics Committee (A5/2011) and in accordance with the Australian Code of Practice for the Care and Use of Animals for Scientific Purposes (NHMRC, 2004). The study has been specifically approved by the CSIRO Animal ethics committee. The steers were fed Flinders grass hay (*Iseilema* spp.) *ad libitum* throughout the study to maintain a consistent microbial activity in the inoculum [34]. Approximately 1 L of rumen liquid and solids were collected from each animal before the morning feed and placed into pre-heated thermal flasks. Pooled rumen fluid was blended at high speed for 30 seconds, using a hand held blender, to ensure complete mixing of solid and liquid phase and detachment of particulate associated bacteria into suspension [35], and then strained through a 1 mm mesh. Strained rumen fluid was continuously purged with high purity N_2_ and maintained at 39°C. Rumen medium was prepared using rumen fluid and pre-heated buffer solution [36] (no trypticase added) in a 1∶4 (vol∶vol) ratio.

A series of batch culture incubations were conducted to assess the effect of species of macroalgae on ruminal fermentation/total gas production and CH_4_ concentration in head-space using an Ankom RF Gas Production System (Ankom Technology, New York, USA). Samples of 0.2 g OM of macroalgae were weighed into pre-warmed 250 mL Schott bottles with 1 g OM of Flinders grass (ground through 1 mm sieve), and 125 mL of rumen medium. To optimize anaerobic conditions, bottles were purged with N_2_, sealed and incubated at 39°C in three temperature controlled incubator/shakers (Ratek, OM11 Orbital Mixer/Incubator, Australia), with the oscillation set at 85 rpm. A positive control bottle containing 1 g OM of Flinders grass and 0.2 g OM of DCS, and a blank containing only rumen medium, were included in each incubator. The incubations were repeated on three different occasions producing a total of four replicates per treatment. For each incubation run, bottles were randomly allocated and placed inside incubators. Each bottle was fitted with an Ankom RF module and monitored for 72 h with reading intervals of 20 minutes to generate TGP curves. Each module contained a pressure valve set to vent at 5 psi. Head-space gas sample were collected from each module directly into pre-evacuated 10 mL exetainers (Labco Ltd, UK) every 24 h. TGP of the head-space sample was converted from pressure readings to mL/g OM.

### Post-fermentation parameters

After 72 h incubation, pH (PHM220 Lab pH Meter, Radiometer Analytical, Lyon, France) was recorded and residual fluid samples were stored at −20°C until analyses. VFAs were quantified at the University of Queensland (Ruminant Nutrition Lab, Galton College, Queensland, Australia) following standard procedures [37], [38], [39]. Total VFA concentration was calculated by subtracting the total VFA concentration in the initial inoculum (buffered rumen fluid) from the total VFA concentration in the residual fluid. Residual fluids were also analysed for total ammonia concentration using semi-automated colorimetry (Tropwater Analytical Services, JCU, Townsville). Solid residues were analysed for apparent degradability of organic matter (OMd), calculated as the proportional difference between organic matter incubated and recovered after 72 h.

CH_4_ concentration in the collected gas samples were measured by gas chromatography (GC-2010, Shimadzu), equipped with a Carbosphere 80/100 column and a Flame Ionization Detector (FID). The temperature of the column, injector and FID were set at 129°C, 390°C, and 190°C, respectively. Helium and H_2_ were used as carrier and burning gases, respectively. Four external standards of known composition: 1) CH_4_ 0% and CO_2_ 0% in N_2_; 2) CH_4_ 3% and CO_2_ 7% in N_2_; 3) CH_4_ 8.89%, CO_2_ 15.4%, and H_2_ 16.8% in N2; and 4) CH_4_ 19.1%, CO_2_ 27.1%, and H_2_ 38.8% in N_2_ (BOC Ltd, Australia) were injected daily for construction of standard curves and used to quantify CH_4_ concentration. Standards were collected following the same procedure used for collection of fermentation gas samples. Additionally, standard 2 (CH_4_ 3% and CO_2_ 7% in N_2_) was injected every 2 h between successive gas samples to verify GC gas composition readings. Head-space samples (1 mL) were injected automatically into the GC to determine CH_4_ concentrations. Peak areas were determined by automatic integration. CH_4_ measured were related to TGP production to estimate relative concentrations [40].

### Data analysis

Corrected TGP data were fitted to a modified non-linear sigmoidal model of Gompertz [41]:
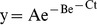
(3)where y is the cumulative total gas production (mL), A the maximal gas production (mL.g^−1^), B the lag period before exponential gas production starts (h), C is the specific gas production rate (mL.h^−1^) at time t (h). The gas production parameters A, B, and C, were calculated using the non-linear procedure of SAS (JMP 10, SAS Institute, Cary, NC, USA). One-way analyses of variance (ANOVA) were used to compare the differences in total gas production (TGP) and CH_4_ production at 72 h between species. Post-hoc comparisons were made using Tukey's HSD multiple comparisons.

Following the ANOVAs, multivariate analyses were used to investigate the relationships between the biochemical and post-fermentation parameters. Two complementary multivariate techniques were used. To examine correlations between variables nonmetric multidimensional scaling was used (MDS; Primer v6 [42]) and to examine possible threshold values for effects Classification and regression tree was used (CART; TreesPlus software, [43]).

For MDS, samples that are close together on plots have similar composition [42]. Thus, a MDS bi-plot was produced to investigate correlations between the biochemical and post-fermentation parameters of species at 72 h incubation. Data was reassembled in a Bray-Curtis similarity matrix using mean values for each species. Information on the strength and nature of the correlation of biochemical or post-fermentation parameters with the distribution of species within the MDS space was represented as vectors in an ordination bi-plot. The parameters most highly correlated with the MDS space, based on Pearson's correlation coefficients (PCC) higher than 0.7, were plotted (Tables 1 and 2).

**Table 1:** Biochemical parameters correlated with MDS and CARTs analyses for TGP and CH_4_ production.

Macroalgae species	Ash	C	GE	H	Total FA	K	N	Sr	PUFA	C 16∶0	Ca	Na	S	Zn
			(MJ kg^−1^ DM)											
**Freshwater algae**														
*Cladophora vagabunda*	158.9	380.2	16.1	57.4	49.6	33.7	54.3	0.03	21.15	8.67	4.2	2.8	11.2	0.02
*Oedogonium* sp.	64.1	447.4	19.4	66.5	57.77	13.3	49.2	0.02	35.14	11.46	2.9	0.4	2.9	0.05
*Spirogyra* sp.	167.7	372.5	15.2	57.6	27.88	5.6	14.7	0.13	16.01	7.39	16.7	38.7	3.1	0.01
**Marine green algae**														
*Caulerpa taxifolia*	269.6	320.2	13.1	48.1	25.5	6.4	32.5	0.07	13.27	7.81	3.8	82.4	22.1	0.01
*Chaetomorpha linum*	254.4	322.3	12.9	48.8	21.09	86.7	42.6	0.05	10.79	5.08	4.5	10	21.4	0.06
*Cladophora coelothrix*	234.1	361.4	15.3	55	30.83	38.6	52.5	0.07	12.67	7.2	7.8	3.9	21	0.03
*Cladophora patentiramea*	365	292.6	11.2	42.1	15.56	60.3	23.9	0.13	4.34	5.18	17.4	3.4	32.8	0.02
*Derbesia tenuissima*	77.5	449.7	20.1	66.3	48.74	9	66.1	0.03	19.16	17.29	2.7	8.2	12.3	0.03
*Ulva* sp.	206.5	322.5	13.6	54.8	25.63	20.5	47.1	0.12	12.6	7.95	10.1	8.4	28.2	0.03
*Ulva ohnoi*	211.3	291.6	12	55.4	14.75	21.6	43	0.05	4.3	5.37	4.5	5.4	57.5	0.04
**Brown algae**														
*Cystoseira trinodis*	266.7	317.3	12.1	46.4	18.69	85.5	18.3	1.23	6.92	6.19	16.3	17.1	13.1	0.01
*Dictyota bartayresii*	300.7	332.8	12.9	46.8	27.01	27	17.9	1.18	9.93	7.15	35.2	5.3	12	0.099
*Hormophysa triquetra*	303.1	296.9	10.7	41.7	18.77	30.8	7.9	0.91	11.15	3.4	21.5	6	13.4	0.06
*Padina australis*	385.6	243.4	8.7	38.6	18.39	81.3	11	1.5	7.73	5.06	21.2	18.4	33.7	0.01
*Sargassum flavicans*	255.8	305	11.7	46.3	13.93	78.1	8.4	1.7	5.67	3.86	20.2	11.7	9.6	0.01
*Colpomenia sinuosa*	409.7	270.6	9.9	38.9	18.3	80.1	14.1	1.5	4.86	5.34	56.3	15.7	7.2	0.05
**Red algae**														
*Asparagopsis taxiformis*	189.4	384	16.4	58.7	27.28	14.7	55.5	0.06	10.13	10.71	6.1	12.8	26.9	0.15
*Halymenia floresii*	277.5	288.5	11.5	48.8	12.97	36.6	21.7	0.07	2.92	6.55	3.9	36	55.7	0.098
*Hypnea pannosa*	473.3	220	7.5	34.9	16.06	19.3	14.3	0.44	6.37	5.16	32.2	54.4	41.6	0.02
*Laurencia filiformis*	359.8	290.7	11.5	44.5	11.99	12.3	18.9	0.31	3.34	4.19	26	64	27.1	0.02
DCS	199	427.8	18.6	64.1	26.51	15.9	79.6	0.01	13.21	6.64	1.9	2.1	3.1	0.05
SEM	0.36	6.66	1.11	0.1	1.29	3.09	0.23	0.74	0.8	0.34	1.49	2.43	1.7	7.35
*r*	0.98	0.98	0.92	0.94	0.81	0.78	0.75	0.76	0.79	0.73	0.7	0.71	0.74	0.21

Parameters were calculated in g.kg^−1^ DM, unless otherwise stated. For TGP and CH_4_ production, (n = 3–4). *r* = Pearson's correlation coefficients from MDS analysis. C, carbon; GE, gross energy content; H, hydrogen, Total FA, total fatty acids; K, potassium; N, nitrogen; Sr, strontium; PUFA, total polyunsaturated fatty acids; C16∶0, palmitic acid; Ca, calcium; Na, sodium; S, sulfur; Zn, zinc; DCS, decorticated cottonseed meal; SEM, standard error mean.

**Table 2:** Post-fermentation parameters correlated with MDS and CARTs analyses for TGP and CH_4_ production.

Macroalgae species	TGP	CH_4_	CH_4_/GP	Volatile Fatty acids (molar proportion)								pH	NH_3_−N	OMd
	(mL.g^−1^ OM)	(mL.g^−1^ OM)	(mL.L^−1^)	Total (mmol/l)	C2	C3	IsoC4	C4	IsoC5	C5	C2∶C3		(mg.L^−1^)	(%)
**Freshwater algae**														
*C. vagabunda*	106.8^abc^	14.3^abc^	133.9	28.52	63.97	26.23	0.73	7.84	0.32	0.91	2.49	6.94	9.00	63.89
*Oedogonium*	101.1^bcd^	12.6^bc^	125.0	32.26	66.42	24.26	0.67	7.28	0.45	0.92	2.79	6.96	7.60	64.50
*Spirogyra*	119.3^ab^	17.3^ab^	144.8	36.59	66.20	23.68	0.45	8.58	0.50	0.58	2.82	6.85	8.20	62.52
**Marine green algae**														
*Caulerpa*	102.3^abcd^	12.2^bc^	119.7	33.46	67.08	23.25	0.58	8.05	0.48	0.57	2.90	6.93	8.60	58.64
*Chaetomorpha*	99.8^bcd^	10.9^bc^	109.3	28.81	62.29	28.84	0.45	7.29	0.24	0.89	2.19	6.97	8.50	60.82
*C coelothrix*	112.6^abc^	13.2^abc^	116.9	27.56	63.79	26.79	0.65	7.46	0.44	0.87	2.39	6.93	8.50	64.20
*C. patentiramea*	79.7^de^	6.1^cde^	76.8	24.29	63.85	26.78	0.45	8.20	0.01	0.71	2.39	7.09	7.80	58.86
*Derbesia*	119.7^ab^	16.3^ab^	136.0	25.18	66.15	24.30	0.78	7.42	0.54	0.81	2.76	6.93	9.40	65.09
*Ulva* sp.	99.0^bcd^	9.0^bcd^	91.1	28.57	63.46	26.68	0.66	7.76	0.47	0.97	2.41	6.99	8.00	61.39
*U. ohnoi*	89.0^cd^	9.9^bcd^	111.6	26.02	65.88	24.45	0.81	7.32	0.62	0.92	2.71	6.95	7.20	61.45
**Brown algae**														
*Cystoseira*	96.8^bcd^	9.9^bc^	102.5	19.64	59.71	32.04	0.10	7.84	0.03	0.29	2.01	6.90	8.10	58.50
*Dictyota*	59.4^ef^	1.4^de^	23.6	17.03	60.94	35.97	0.06	2.81	0.00	0.23	1.73	7.13	7.90	58.09
*Hormophysa*	104.8^abcd^	10.2^bc^	97.0	21.24	64.98	28.07	0.14	6.39	0.04	0.37	2.37	6.93	7.70	62.05
*Padina*	97.4^bcd^	9.0^cd^	92.4	24.56	65.25	26.00	0.35	7.49	0.19	0.72	2.53	6.97	7.00	60.00
*Sargassum*	113.6^abc^	11.9^bc^	105.0	29.23	66.47	24.40	0.45	8.03	0.27	0.38	2.77	6.89	7.70	60.79
*Colpomenia*	95.8^bcd^	9.2^bcd^	95.5	23.06	62.70	29.08	0.30	7.50	0.00	0.29	2.16	6.99	8.10	61.84
**Red algae**														
*Asparagopsis*	48.4^f^	0.2^e^	4.3	14.79	39.96	40.23	0.00	19.27	0.00	0.54	0.92	7.08	6.70	59.26
*Halymenia*	114.0^abc^	13.3^abc^	116.3	22.52	64.67	23.95	0.83	8.96	0.65	0.94	2.71	6.91	8.30	61.42
*Hypnea*	101.9^abcd^	10.4^bc^	102.1	28.44	66.62	23.99	0.58	7.77	0.41	0.63	2.78	6.96	6.70	60.85
*Laurencia*	96.1^bcd^	10.9^bc^	113.0	24.36	65.73	25.36	0.33	8.12	0.08	0.37	2.59	6.95	7.70	61.17
DCS	126.8^a^	18.1^a^	142.9	27.80	64.00	25.53	0.80	7.89	0.63	1.16	2.55	6.91	9.50	64.51
SEM	2.29	0.61	4.60	0.94	0.75	0.63	0.37	0.31	0.04	0.04	0.06	0.01	0.11	0.49
*r*	0.19	0.42	0.34	0.37	0.23	0.34	0.43	0.17	0.62	0.45	0.35	0.19	0.59	0.55

For TGP and CH4 production, (n = 3–4) species not connected by the same letters within the same column are significantly different.

*r* = Pearson's correlation coefficients from MDS analysis; C2, acetate; C3, propionate; C4, butyrate; Iso C4, Iso-butyrate; C5, valerate; Iso C5, Iso -valerate C2∶C3, acetate/propinate ratio; OMd, organic matter degraded; DCS, decorticated cottonseed meal; SEM, standard error mean.

Because there were no overarching relationships between the major primary compositional variables and TGP, CH_4_, and other post-fermentation variables (see Results), a multivariate CART was conducted to test the direct effects of biochemical compositional values for each species on TGP, CH_4_ production, acetate and propionate concentrations [43]. In this instance, CART was used to highlight independent variables that may have subtle or interactive effects on the post-fermentation parameters. Data was fitted using 10-fold cross validation based on minimizing the error sum of squares [43], [44]. The sum of squares is equivalent to the least squares of linear models [44]. Final tree models were chosen based on the ±1SE rule [44], [45], which provided 2 key independent variables for the split.

## Results

### Total gas and methane production

**Figure 2:**
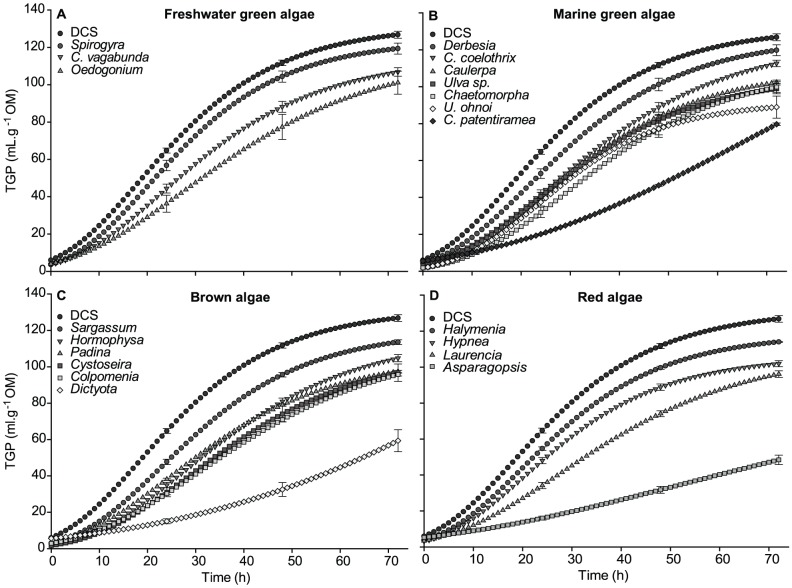
Total gas production of macroalgae species over the 72 h incubation period. Error bars represent ±SE (n = 4). Species full names are given in Table 1.

Total gas production (TGP) was lower for all species of macroalgae compared to DCS (Fig. 2, ANOVA: 72 h, F_20,63_ = 14.36, p<0.001). The freshwater green macroalga *Spirogyra* (Fig. 2a) and the marine green macroalga *Derbesia* (Fig. 2b) had the highest TGP of all species, producing a total of 119.3 mL.g^−1^ OM and 119.7 mL.g^−1^ OM, respectively, and were not significantly different from DCS (Table 2, Tukey's HSD 72 h, p>0.05). *Oedogonium* was the only freshwater green macroalga that was significantly different from DCS (Fig. 2a, Tukey's HSD 72 h, p<0.05), decreasing TGP by up to 20.3% after 72 h incubation. *Cladophora patentiramea* had the lowest TGP of the marine green macroalgae, producing a total of 79.7 mL.g^−1^ OM (Fig. 2b). The effect was most prominent at 24 h when TGP was reduced by 68.9% compared to DCS, and TGP was significantly reduced at 72 h, (Fig. 2b, Tukey's HSD 72 h, p<0.0001). *Dictyota* was the most effective species of brown macroalgae, reducing TGP to 59.4 mL.g^−1^ OM after 72 h (Fig. 2c), resulting in a significantly lower TGP (53.2%) than for DCS (Fig. 2c, Tukey's HSD 72 h, p<0.0001). This effect was even greater at 24 h (TGP = 76.7% lower than DCS). Although other brown macroalgae were not as effective as *Dictyota*, overall they reduced TGP by at least >10%, with *Padina*, *Cystoseira*, and *Colpomenia* significantly reducing TGP compared to DCS (Table 2, Tukey's HSD 72 h, p<0.02). The most effective of all macroalgae was the red alga *Asparagopsis* (Fig. 2d) with the lowest TGP, 48.4 mL.g^−1^ OM. Although *Asparagopsis* had a similar trend to *Dictyota* for the first 48 h, its efficacy was maintained throughout the incubation period, producing 61.8% less TGP than DCS after 72 h.

**Figure 3:**
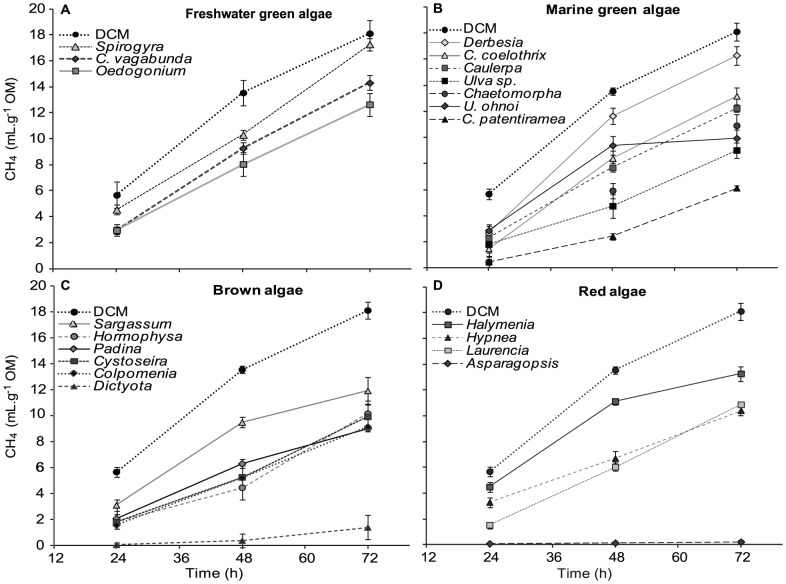
Methane production of macroalgae species at 24, 48, and 72 h. Error bars represent ±SE (n = 3–4). Species full names are given in Table 1.

CH_4_ production generally followed the same pattern as TGP and notably CH_4_ production was directly and significantly correlated with TGP values (Figure S1). DCS had the highest CH_4_ output, producing 18.1 mL.g^−1^ OM at 72 h. All macroalgal treatments were, on average, lower than DCS after 72 h (Fig. 3, ANOVA: 72 h, F_20,55_ = 10.24, p<0.0001). In a similar manner to TGP, the freshwater green macroalga *Spirogyra* (Fig. 3a) and marine green macroalga *Derbesia* (Fig. 3b) had the highest CH_4_ production of all species, and grouped with DCS (Table 2, Tukey's HSD 72 h, p>0.05). *Asparagopsis*, *Dictyota* and *C. patentiramea* also had the most pronounced effect on reducing *in vitro* CH_4_ production. *C. patentiramea* had a CH_4_ output of 6.1 mL.g^−1^ OM (Table 1) and produced 66.3% less CH_4_ than DCS (Fig. 3b, Tukey's HSD 72 h, p<0.0001). *Dictyota* produced 1.4 mL.g^−1^ OM and was the most effective of the brown macroalgae, reducing CH_4_ output by 92% (Fig. 3c, Table 2, Tukey's HSD 72 h, p<0.001), and the concentration of CH_4_ within TGP, 23.6 mL.L^−1^, by 83.5% compared to DCS (Table 2). *Asparagopsis* had the lowest CH_4_ output among all species of macroalgae producing a maximum of 0.2 mL.g^−1^ OM throughout the incubation period (Table 2, Tukey's HSD 72 h, p<0.001). This is a reduction of 98.9% on CH_4_ output compared to DCS (Fig. 3d), independently of time. Notably, *Asparagopsis* also had the lowest concentration of CH_4_ within TGP producing only 4.3 mL.L^−1^ of CH_4_ per litre of TGP after 72 h, making it distinct from all other species (Table 2).

### Other post-fermentation parameters

There were significant effects of macroalgae on VFA production among species (ANOVA: 72 h, F_20,60_ = 2.01, p = 0.02). *Spirogyra* produced 36.59 mmol.L^−1^ of VFA, the highest total VFA production among all species and 31.6% more than DCS. *Oedogonium*, *C. vagabunda*, *Caulerpa*, *Chaetomorpha*, *Ulva* sp., *Sargassum* and *Hypnea* also produced 2.3% to 20.4% more VFA than the control DCS (Table 2). *Dictyota* and *Asparagopsis* had the lowest total VFA production. The decrease in total VFA was influenced by the inhibition of acetate (C2) production leading to a decrease in the C2∶C3 ratio. *Asparagopsis* had the lowest C2∶C3 ratio, 0.92, followed by *Dictyota* with almost double this value, 1.73 (Table 2).

Ammonia (NH_3_) production varied significantly among species (ANOVA: 72 h, F_20,63_ = 3.37, p<0.0001). DCS had the highest concentration of NH_3_ at 9.5 mg N.L^−1^, while *Asparagopsis* and *Hypnea* had the lowest NH_3_ concentration of 6.7 mg N.L^−1^. Although apparent organic matter degradability (OMd) varied from a minimum of 58% for *Dictyota* to maximum of 64% for DCS, this difference was not significant (p>0.05). Similarly pH varied from a minimum of 6.85 for *Spirogyra* to a maximum of 7.13 for *Dictyota* (Table 2), this difference was not significant and all values were within the range required to maximize fiber digestion for ruminant.

### Biochemical and post-fermentation parameters

**Figure 4:**
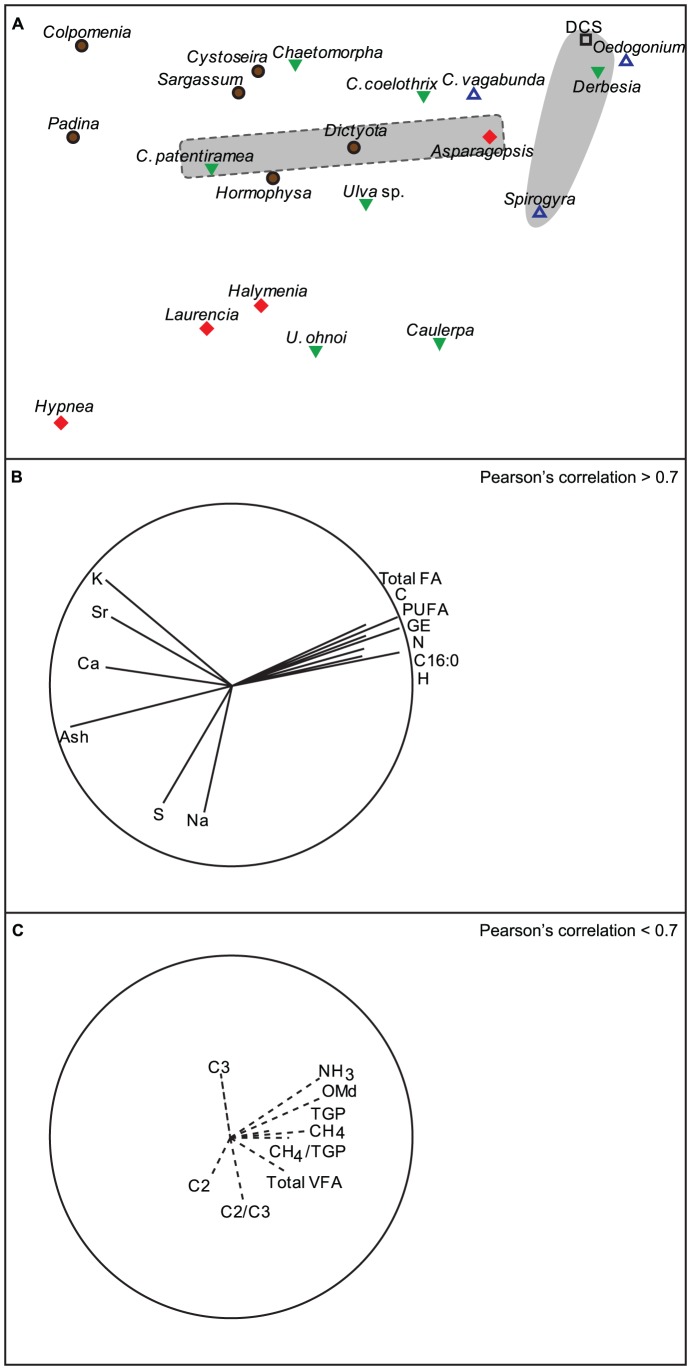
MDS showing similarities between macroalgae species based on biochemical and post-fermentation parameters. (A) MDS plot (Stress = 0.11) of the distribution of species within ordination space. Species within grey cluster had the highest TGP and CH_4_ production, while species within dotted line grey cluster had the lowest TGP and CH_4_ production. (B) MDS vectors with Pearson's correlation coefficients (*r*) higher than 0.7 superimposed. (C) Post-fermentation parameters vectors superimposed (note all correlation coefficients lower than 0.7, see Table 2). White and blue triangles: Freshwater green algae, green triangles: Marine green algae, brown circles: Brown algae, red diamonds: Red algae, and square: DCS. Species full names are given in Table 1.

The MDS bi-plot between biochemical parameters and post-fermentation parameters at 72 h showed that *Oedogonium* and *Derbesia* grouped closely with DCS, and this grouping was most similar to *C. vagabunda*, *C. coelothrix*, *Asparagopsis* and *Spirogyra* (Fig. 4a). The biochemical parameters with the highest correlation with the MDS space were ash, C, GE, and H and these were the most important parameters in differentiating algae (Table 1). The species located on the top right corner of the MDS bi-plot (Fig. 4a) were positively correlated to the elements C, N, H, and GE, total fatty acid, polyunsaturated fatty acid (PUFA) and C∶16 (Fig. 4b). Most brown macroalgae grouped together on the top left corner of the MDS plot (Fig. 4a) with *Padina*, *Colpomenia*, and *Sargassum* having the highest Strontium concentrations of >1.5 g.kg^−1^ DM (Table 1). Species with higher TGP and CH_4_ production clustered on the left side of the MDS bi-plot (continuous line cluster, Fig. 4a). However, species with low TGP and CH_4_ production were spread across the bi-plot (dotted line cluster, Fig. 4a), indicating that these variables were not strongly correlated to any of the main biochemical variables that affected the spread of species within the MDS (*r*<0.19, and 0.42, respectively; Fig. 4a). Similarly, the other post-fermentation parameters were not strongly correlated to any biochemical parameter in the MDS bi-plot (Fig. 4c, Table 2).

**Figure 5:**
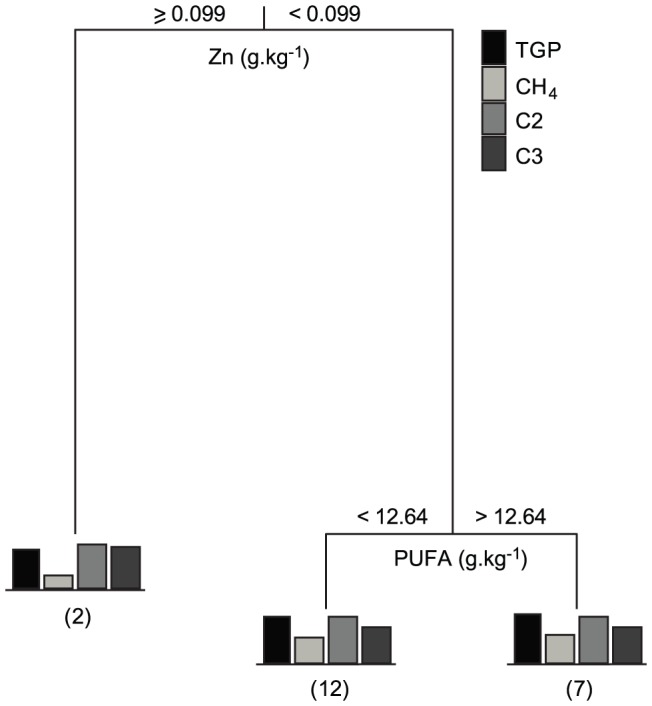
Multivariate classification and regression tree model. This CART is based on biochemical variables explaining 79.1% of the variability in total gas production (TGP), CH_4_ production, and acetate (C2) and propionate (C3) molar proportions. Data was fourth-root transformed. Numbers in brackets indicate the number of species grouped in each terminal branch.

A multivariate CART model was produced to investigate the direct effects of biochemical parameters on the main fermentation parameters, TGP, CH_4_ production, acetate and propionate concentrations (Fig. 5). The best tree model, explaining 79.1% of the variability in the data, showed that zinc was the independent variable with the highest relative importance (100%), splitting *Asparagopsis* and *Dictyota*, which had a concentration of zinc ≥0.099 g.kg^−1^ DM, from the remaining species (Table 1). These two species had the lowest TGP and CH_4_ production and the highest proportion of propionate. However, *Halymenia* had a similar concentration of zinc, 0.099 g.kg^−1^ DM and the highest TGP and CH_4_ output of any species of red and brown macroalgae (Table 1). This suggests that a zinc threshold is interacting with other biochemical variables, specific to *Asparagopsis* and/or *Dictyota*, which affects these fermentation parameters. The lack of a linear relationship is also confirmed by the low correlation of zinc with the MDS space (*r* = 0.21). For species with a concentration of zinc <0.099 g.kg^−1^ DM, differences in polyunsaturated fatty acid (PUFA) concentration generated a second split, indicating that species with PUFA>12.64 g.kg^−1^ DM had higher CH_4_ production than species with PUFA concentration below this value. However, PUFA had a relative importance of 14.8% of zinc indicating that the influence of PUFA in the model was small.

## Discussion

While the nutritional manipulation of enteric methane production using terrestrial plants/forages has been extensively investigated [6], [11], [46], [47], this study provides the first evidence that macroalgae can effectively reduce *in vitro* methane production as all species had similar or lower TGP and CH_4_ production to a positive control of decorticated cottonseed (DCS). Importantly, cottonseed is used as a feed supplement for cattle because it considerably reduces CH_4_ production compared to other high energy grains [5], [46], [48]. The reduction in total gas production, compared to DCS, was similar among species, with the exception of *Asparagopsis*, *Dictyota* and *C. patentiramea* which were most effective.

In general, marine algae were more effective than freshwater algae in reducing CH_4_ production. Freshwater macroalgae have a similar biochemical composition to DCS, however, the CH_4_ output relative to DCS was reduced to 4.4% for *Spirogyra* and 30.3% for *Oedogonium* after 72 h incubation. However, there is no correlation between the biochemical composition of freshwater and a reduction in CH_4_. Although CH_4_ was reduced there were no apparent negative effects on fermentation variables. Rather, freshwater macroalgae had slightly higher total VFA concentration than DCS with similar organic matter degradability (OMd), demonstrating that fermentation processes had not been compromised [49].

Marine algae reduced CH_4_ production significantly, with two species, the brown macroalga *Dictyota* and the red macroalga *Asparagopsis* having the most significant effects. *Dictyota* inhibited TGP by 53.2% and CH_4_ production by over 92% compared to DCS, while *Asparagopsis* was the most effective treatment reducing TGP by 61.8%, and CH_4_ production by 98.9% compared to DCS. *Dictyota* and *Asparagopsis* also produced the lowest total VFA concentration and the highest molar concentration of propionate among all species, demonstrating that fermentation was significantly affected. A decrease in the concentration of total VFAs is often associated with anti-nutritional factors that interfere with ruminal fermentation [49]. *Asparagopsis*, at the concentrations tested, was over 17 times more effective in reducing the proportion of CH_4_ within total gas produced than terrestrial plants high in tannins [50], or some feed cereals or legumes [51]. *Asparagopsis* has a similar (primary) biochemical composition to DCS with the exception of high levels of zinc and low PUFA. Both *Asparagopsis* and *Dictyota* had high concentration of zinc, however, *Halymenia* also had a similar concentration but produced 47.9% more TGP and 89.5% more CH_4_ than *Dictyota*. Notably, when zinc is added to a diet at a concentration above 250 mg.Kg^−1^ DM, it can reduce *in vitro* substrate degradability and increase molar proportion of propionate [52], which are indicative parameters of reduced methane output. However, the concentration of zinc in *Dictyota* was 0.099 mg.Kg^−1^ DM and in *Asparagopsis* 0.15 mg.Kg^−1^ DM, and these concentrations are far below the threshold of 250 mg.Kg^−1^ DM. Therefore, there is little supporting evidence that zinc reduces the production of CH_4_ to the extent to which it occurs in *Dictyota* and *Asparagopsis*. It is possible, however, that zinc acts synergistically with secondary metabolites produced by both species of algae to reduce CH_4_ production. Some elements can enhance secondary metabolite concentrations of plants even at low threshold concentrations [53]. Both *Asparagopsis* and *Dictyota* are rich in secondary metabolites with strong antimicrobial properties [54] and the lack of a strong relationship between gas and methane production, and any of the >70 primary biochemical parameters analysed, suggests that the reduction in total gas production and CH_4_ is associated with secondary metabolites.

Secondary metabolites function as natural defences against predation, fouling organisms and microorganisms, and competition among species [55]. There is an increasing interest on these secondary metabolites due to their anti-microbial, anti-fungal, and anti-viral activities [56]. *Dictyota* produces an array of secondary metabolites, in particular, isoprenoids (terpenes) [56]. *Asparagopsis* produces halogenated low molecular weight compounds, in particular brominated and chlorinated haloforms [54], [57]. Many of these compounds have strong antimicrobial properties and inhibit a wide range of microorganisms, including Gram-positive and Gram-negative bacteria, as well as, mycobacterium and fungus activities [54], [56], [58]. Secondary metabolites from *Asparagopsis* also inhibit protozoans [59]. Given the significant effects of *Asparagopsis* in reducing total gas production and CH_4_ output, it is likely that lower doses of this alga can now be targeted to reduce CH_4_ output without affecting the nutritionally important fermentation parameters.

## Conclusions

This study provides an extensive quantitative and qualitative assessment of tropical macroalgae to identify suitable species for the mitigation of enteric CH_4_ emissions. All species demonstrated potential for this purpose, producing less CH_4_ than DCS. *Dictyota* and *Asparagopsis* were the most promising species reducing CH_4_ output by 92.2% and 98.9% respectively, after 72 h incubation. However, these species also affected fermentation, decreasing the total VFA concentration. Due to their effectiveness, it is likely that lower concentration can inhibit CH_4_ production and minimize their effects on anaerobic fermentation. In contrast, other species, in particular freshwater macroalgae, may decrease methane output at higher doses and maintain nutritional equivalency to traditional feed components. Further, studies are under way to identify the optimum concentration and algae combinations that will reduce CH_4_ without affecting fermentation and eventually evaluate the reduction of enteric methane by macroalgae *in vivo*.

## Supporting Information

Table S1 **Proximate analysis of freshwater and marine macroalgae species, decorticated cottonseed meal (DCS) and Flinders grass hay.**
(DOCX)

Table S2 **Elemental analysis (±SD) of freshwater and marine macroalgae species, decorticated cottonseed meal (DCS) and Flinders grass hay (mg.Kg^−1^ DM).**
(DOCX)

Table S3 **Fatty acid profiles (±SD) of macroalgae species, decorticated cottonseed meal (DCS) and Flinders grass hay.**
(DOCX)

Figure S1 **Linear relationship between total gas and CH_4_ production for macroalgae species and decorticated cottonseed meal.** Individual data points represent mean values (mg.g^−1^ OM, ± SE) for each species. Function is only predictive within the shown data range.
(EPS)
