# Supplementary material for: Effects of Marine and Freshwater Macroalgae on In Vitro Total Gas and Methane Production
Source: PLoS One. 2014 Jan 22;9(1):e85289. doi: 10.1371/journal.pone.0085289 (PMC3898960; doi:10.1371/journal.pone.0085289)
Supplement: Table S1 — Proximate analysis of freshwater and marine macroalgae species, decorticated cottonseed meal (DCS) and Flinders grass hay. (DOCX) [file pone.0085289.s001.docx]

| **Species** | **Site** | **FW:DW** | **DM** | **OM** | **CP** | **TL** | **Carbohydrates** | **Ash** | **GE (MJ.kg^-1^ DM)** |
| --- | --- | --- | --- | --- | --- | --- | --- | --- | --- |
| **Freshwater algae** |  |  |  |  |  |  |  |  |  |
| *Cladophora vagabunda* | MARFU^A^ | 6.31 | 940.87 | 841.11 | 278.56 | 96.76 | 406.66 | 158.89 | 16.08 |
| *Oedogonium* sp. | MARFU | 4.37 | 937.93 | 935.90 | 252.40 | 79.35 | 542.08 | 64.10 | 19.41 |
| *Spirogyra* sp. | GFB^B^ Kelso | 11.98 | 926.84 | 832.35 | 75.41 | 52.09 | 631.69 | 167.65 | 15.18 |
|  |  |  |  |  |  |  |  |  |  |
| **Green algae** |  |  |  |  |  |  |  |  |  |
| *Caulerpa taxifolia* | MARFU | 11.11 | 930.81 | 730.39 | 166.73 | 58.98 | 435.49 | 269.61 | 13.07 |
| *Chaetomorpha linum* | MARFU | 6.00 | 934.81 | 745.56 | 218.54 | 47.89 | 413.94 | 254.44 | 12.86 |
| *Cladophora coelothrix* | GFB^B^ Bowen | 3.72 | 923.57 | 765.90 | 269.33 | 49.96 | 370.18 | 234.10 | 15.32 |
| *Cladophora patentiramea* | PR^C^ | 4.45 | 938.31 | 635.04 | 122.61 | 26.07 | 424.67 | 364.96 | 11.22 |
| *Derbesia tenuissima* | MARFU | 8.10 | 919.27 | 922.52 | 339.09 | 130.13 | 372.55 | 77.48 | 20.14 |
| *Ulva* sp. | MARFU | 6.90 | 911.42 | 793.49 | 241.62 | 33.05 | 430.23 | 206.51 | 13.57 |
| *Ulva ohnoi* | MARFU | 6.52 | 907.00 | 788.74 | 220.59 | 24.56 | 450.59 | 211.26 | 12.02 |
|  |  |  |  |  |  |  |  |  |  |
| **Brown algae** |  |  |  |  |  |  |  |  |  |
| *Cystoseira trinodis* | NB^D^ | 6.39 | 919.95 | 733.33 | 98.45 | 35.22 | 524.18 | 266.67 | 12.09 |
| *Dictyota bartayresii* | NB and RB^E^ | 6.74 | 945.44 | 699.27 | 96.30 | 112.82 | 440.06 | 300.73 | 12.86 |
| *Hormophysa triquetra* | NB | 5.73 | 925.32 | 696.93 | 42.50 | 33.94 | 547.78 | 303.07 | 10.68 |
| *Padina australis* | RB | 5.38 | 933.88 | 614.43 | 59.18 | 24.98 | 466.90 | 385.57 | 8.65 |
| *Sargassum flavicans* | NB | 6.80 | 925.19 | 744.19 | 45.19 | 27.21 | 599.08 | 255.81 | 11.67 |
| *Colpomenia sinuosa* | NB | 15.63 | 945.06 | 590.31 | 75.86 | 31.05 | 431.99 | 409.69 | 9.86 |
|  |  |  |  |  |  |  |  |  |  |
| **Red algae** |  |  |  |  |  |  |  |  |  |
| *Asparagopsis taxiformis* | MARFU | 3.73 | 944.82 | 810.58 | 254.75 | 33.33 | 437.35 | 189.42 | 16.44 |
| *Halymenia floresii* | NB | 7.88 | 929.30 | 722.50 | 99.60 | 15.14 | 525.34 | 277.50 | 11.55 |
| *Hypnea pannosa* | NB | 10.40 | 935.74 | 526.65 | 65.64 | 28.51 | 360.52 | 473.35 | 7.54 |
| *Laurencia filiformis* | NB | 11.70 | 936.57 | 640.21 | 86.75 | 64.32 | 415.50 | 359.79 | 11.46 |
|  |  |  |  |  |  |  |  |  |  |
| DCS | - | - | 897.91 | 801.01 | 497.50 | 47.18 | 154.24 | 198.99 | 18.55 |
| Flinders grass | - | - | 925.92 | 875.76 | 27.50 | 28.68 | 745.51 | 124.24 | 15.51 |

^A^ Marine and Aquaculture Research Facility Unit, Macroalgal Biofuels and Bioproducts Research Group, James Cook University (19.33^o^S; 146.76^o^E); ^B^ Good Fortune Bay Fisheries, a barramundi farm (19.36^o^S; 146.70^o^E); ^C^ Pacific Reef Fisheries, Tiger prawn farm (19.58°S, 147.40°E); ^D^ Nelly Bay, an intertidal reef flat situated in Magnetic Island (19.16°S; 146.85°E), ^E^ Rowes Bay, an intertidal reef flat situated in Townsville (19.23°S, 146.79°E).

Parameters were calculated in g.kg^-1^ DM, unless otherwise stated; FW:DW, fresh weight to dry weight ratio; DM, dry matter; OM, organic matter; CP, crude protein (nitrogen factors of 5.13, 5.38, and 4.59 for green, brown and red macroalgae, respectively [25], and 6.25 for cottonseed and Flinders grass hay); TL, total lipids; GE, gross energy; (n = 2 ).
