# Supplementary material for: Effects of Marine and Freshwater Macroalgae on In Vitro Total Gas and Methane Production
Source: PLoS One. 2014 Jan 22;9(1):e85289. doi: 10.1371/journal.pone.0085289 (PMC3898960; doi:10.1371/journal.pone.0085289)
Supplement: Table S2 — Elemental analysis (±SD) of freshwater and marine macroalgae species, decorticated cottonseed meal (DCS) and Flinders grass hay (mg.Kg−1 DM). (DOCX) [file pone.0085289.s002.docx]

| **Species** | **Al** | **As*** | **B** | **Ba** | **C** | **Ca**^ | **Cd*** | **Co**^ | **Cr**^ |
| --- | --- | --- | --- | --- | --- | --- | --- | --- | --- |
| **Freshwater green algae** |  |  |  |  |  |  |  |  |  |
| *C vagabunda* | 109 ± 1 | 1.1 ± 0.02 | 46.4 ± 1.1 | 64.1 ± 1.1 | 380193 ± 44 | 4150 ± 29 |  | 0.35 ± 0.003 | 0.7 ± 0.04 |
| *Oedogonium* | 307 ± 2 |  | 2.7 ± 0.5 | 54.2 ± 1.3 | 447447 ± 5 | 2850 ± 15 |  | 0.53 ± 0.005 | 1.4 ± 0.04 |
| *Spirogyra* | 770 ± 8 | 10.4 ± 0.2 | 3.9 ± 0.45 | 2420 ± 69 | 372454 ± 419 | 16700 ± 157 | 0.08 ± 0.003 | 0.89 ± 0.014 | 0.1 ± 0.02 |
|  |  |  |  |  |  |  |  |  |  |
| **Marine green algae** |  |  |  |  |  |  |  |  |  |
| *Caulerpa* | 34.1 ± 4.8 | 1.0 ± 0.04 | 18.5 ± 0.8 | 6.7 ± 0.16 | 320232 ± 2128 | 3750 ± 13 | 0.06 ± 0.002 | 0.17 ± 0.002 | 0.3 ± 0.02 |
| *Chaetomorpha* | 68.4 ± 1.9 | 2.0 ± 0.04 | 176 ± 2 | 2.7 ± 0.02 | 322278 ± 2190 | 4540 ± 18 | **0.49 ± 0.014** | 0.28 ± 0.005 | 0.3 ± 0.01 |
| *Cladophora* | 2580 ± 25 | 7.0 ± 0.13 | 292 ± 4 | 17.2 ± 0.2 | 361389 ± 990 | 7790 ± 36 | 0.11 ± 0.002 | 1.39 ± 0.01 | 2.6 ± 0.05 |
| *C. patentiramea* | 3320 ± 18 | 3.7 ±0.05 | 212 ± 2 | 26.6 ± 0.4 | 292572 ± 2316 | 17400 ± 182 | 0.18 ± 0.002 | 4.36 ± 0.05 | 3.7 ± 0.1 |
| *Derbesia* | 55 ± 3.7 | 5.5 ± 0.12 | 43 ± 1.4 | 3.2 ± 0.03 | 449668 ± 2616 | 2740 ± 19 | 0.29 ± 0.009 | 0.67 ± 0.012 | 0.3 ± 0.03 |
| Ulva sp. | 470 ± 6 |  | 591 ± 5 | 6.0 ± 0.07 | 322491 ± 2200 | 10100 ± 100 | **0.48 ± 0.004** | 0.34 ± 0.005 | 1.1 ± 0.04 |
| *U. ohnoi* | 24.9 ± 2.2 |  | 61.6 ± 2.3 | 2.7 ± 0.05 | 291623 ± 1274 | 4540 ± 34 | 0.24 ± 0.006 | 0.48 ± 0.013 | 0.9 ± 0.03 |
|  |  |  |  |  |  |  |  |  |  |
| **Brown algae** |  |  |  |  |  |  |  |  |  |
| *Cystoseira* | 1120 ± 10 | **148 ± 3** | 125 ± 2 | 13.9 ± 0.2 | 317347 ± 1114 | 16300 ± 164 | **0.41 ± 0.009** | 0.52 ± 0.011 | 0.6 ± 0.31 |
| *Dictyota* | 6890 ± 78 | 20.4 ± 0.3 | 136 ± 5 | 28.2 ± 0.8 | 332795 ± 2976 | 35200 ± 177 | **1.25 ± 0.02** | 1.38 ± 0.04 | 3.8 ± 0.03 |
| *Hormophysa* | 6860 ± 77 | 16.5 ± 0.3 | 55.4 ± 0.9 | 33.5 ± 0.6 | 296874 ± 3371 | 21500 ± 100 | 0.18 ± 0.005 | 1.09 ± 0.01 | 3.5 ± 0.05 |
| *Padina* | 1640 ± 26 | **79.5 ± 1.6** | 102 ± 1 | 17.2 ± 0.1 | 243383 ± 541 | 21200 ± 273 | 0.09 ± 0.001 | 0.36 ± 0.005 | 1 ± 0.02 |
| *Sargassum* | 1230 ± 20 | **54.5 ± 1.1** | 149 ± 2 | 18 ± 0.2 | 305020 ± 560 | 20200 ± 100 | **0.51 ± 0.014** | 0.61 ± 0.008 | 0.8 ± 0.03 |
| *Colpomenia* | 13200 ± 106 | 18.2 ± 0.3 | 28.2 ± 1.7 | 35.4 ± 0.4 | 270564 ± 1057 | 56300 ± 364 | 0.10 ± 0.005 | 1.49 ± 0.04 | 5.9 ± 0.13 |
|  |  |  |  |  |  |  |  |  |  |
| **Red algae** |  |  |  |  |  |  |  |  |  |
| *Asparagopsis* | 360 ± 1 | 2.8 ± 0.05 | 159 ± 4 | 3.9 ± 0.04 | 383998 ± 598 | 6050 ± 34 | **0.52 ± 0.005** | 0.23 ± 0.005 | 0.6 ± 0.03 |
| *Halymenia* | 40.6 ± 1.5 | 16.9 ± 0.3 | 59.4 ± 1.2 | 0.9 ± 0.01 | 288515 ± 1153 | 3910 ± 30 | **2.79 ± 0.06** | 2.09 ± 0.04 | 0.2 ± 0.02 |
| *Hypnea* | 6660 ± 35 | 9.5 ± 0.16 | 149 ± 4 | 17 ± 0.3 | 219976 ± 1674 | 32200 ± 450 | 0.33 ± 0.008 | 1.02 ± 0.01 | 4.2 ± 0.11 |
| *Laurencia* | 5200 ± 60 | 10.7 ± 0.3 | 114 ± 3 | 9.8 ± 0.13 | 290681 ± 1558 | 26000 ± 196 | 0.31 ± 0.007 | 0.71 ± 0.012 | 2.8 ± 0.08 |
|  |  |  |  |  |  |  |  |  |  |
| DCS | 2.1 ± 0.1 |  | 23.5 ± 1.4 | 1.5 ± 0.03 | 427763 ± 1922 | 1850 ± 18 |  | 0.43 ± 0.016 |  |
| Flinders grass | 759 ± 3 |  | 9.6 ± 0.5 | 16.6 ± 0.2 | 389407 ± 1560 | 3490 ± 36 |  | 0.19 ± 0.001 | 0.8 ± 0.02 |
|  |  |  |  |  |  |  |  |  |  |

**Table S2 –** Continuation.

| **Species** | **Cu**^ | **Fe**^ | **H** | **K**^ | **Mg**^ | **Mn**^ | **Mo**^ | **N** | **Na**^ |
| --- | --- | --- | --- | --- | --- | --- | --- | --- | --- |
| **Freshwater green algae** |  |  |  |  |  |  |  |  |  |
| *C vagabunda* | 8.2 ± 0.18 | **930 ± 7** | 57363 ± 174 | **33700 ± 104** | 2110 ± 13 | 578 ± 4 | **9.5 ± 0.1** | 54296 ± 742 | 2790 ± 10 |
| *Oedogonium* | 55.8 ± 2.1 | **1860 ± 16** | 66547 ± 477 | 13300 ± 109 | 2140 ± 49 | 180 ± 3 | 2.1 ± 0.07 | 49219 ± 115 | 424 ± 7 |
| *Spirogyra* | 4.0 ± 0.12 | 385 ± 1 | 57617 ± 1139 | 5640 ± 62 | 3110 ± 43 | **1320 ± 18** | 0.8 ± 0.03 | 14719 ± 419 | 38700 ± 604 |
|  |  |  |  |  |  |  |  |  |  |
| **Marine green algae** |  |  |  |  |  |  |  |  |  |
| *Caulerpa* | 2.2 ± 0.04 | 40.6 ± 0.1 | 48077 ± 84 | 6390 ± 38 | **5800 ± 10** | 5.3 ± 0.1 | 0.9 ± 0.08 | 32478 ± 17 | 82400 ± 806 |
| *Chaetomorpha* | 21.3 ± 0.4 | 474 ± 3 | 48794 ± 447 | **86700 ± 316** | **6220 ± 69** | 30.9 ± 0.6 | 1.5 ± 0.24 | 42552 ± 440 | 9950 ± 39 |
| *Cladophora* | **93.8 ± 2.4** | **3390 ± 28** | 55033 ± 244 | **38600 ± 351** | **5320 ± 50** | 92.5 ± 1 | 2.2 ± 0.05 | 52462 ± 144 | 3850 ± 23 |
| *C. patentiramea* | 10.1 ± 0.1 | **4350 ± 11** | 42131 ± 1063 | 60300 ± 537 | **4990 ± 49** | **5480 ± 90** | 2.1 ± 0.13 | 23887 ± 1183 | 3430 ± 38 |
| *Derbesia* | 22.5 ± 0.5 | **1990 ± 10** | 66253 ± 1063 | 8990 ± 27 | **5050 ± 47** | 55.4 ± 0.9 | 0.8 ± 0.01 | 66072 ± 130 | 8180 ± 74 |
| Ulva sp. | 31 ± 0.5 | 766 ± 11 | 54847 ± 378 | 20500 ± 100 | **26700 ± 497** | 34.5 ± 0.5 | 0.6 ± 0.01 | 47075 ± 494 | 8430 ± 188 |
| *U. ohnoi* | 11.4 ± 0.2 | 110 ± 1 | 55415 ± 258 | 21600 ± 290 | **37800 ± 100** | 10.0 ± 0.4 | 0.4 ± 0.02 | 43018 ± 227 | 5390 ± 74 |
|  |  |  |  |  |  |  |  |  |  |
| **Brown algae** |  |  |  |  |  |  |  |  |  |
| *Cystoseira* | 1.3 ± 0.04 | 698 ± 3 | 46413 ± 247 | **85500 ± 1960** | **7830 ± 52** | 26.4 ± 0.2 | 1.2 ± 0.08 | 18332 ± 352 | 17100 ± 105 |
| *Dictyota* | 6.9 ± 0.16 | **4600 ± 14** | 46808 ± 554 | 27000 ± 164 | **27000 ± 181** | 458 ± 5 | 1.1 ± 0.07 | 17917 ± 683 | 5310 ± 33 |
| *Hormophysa* | 9.2 ± 0.11 | **4420 ± 39** | 41653 ± 217 | **30800 ± 429** | **10900 ± 100** | 179 ± 2 | 1.1 ± 0.02 | 7897 ± 183 | 6010 ± 72 |
| *Padina* | 3.1 ± 0.06 | **997 ± 13** | 38562 ± 88 | **81300 ± 138** | **6810 ± 35** | 27 ± 0.5 | 1.3 ± 0.22 | 10966 ± 438 | 18400 ± 100 |
| *Sargassum* | 3.0 ± 0.07 | 801 ± 5 | 46314 ± 404 | **78100 ± 1060** | **7010 ± 80** | 59.7 ± 0.5 | 1.7 ± 0.16 | 8430 ± 64 | 11700 ± 199 |
| *Colpomenia* | 7.0 ± 0.15 | **8150 ± 19** | 38868 ± 538 | **80100 ± 1520** | **7480 ± 72** | 156 ± 2 | 1.3 ± 0.02 | 14067 ± 258 | 15700 ± 112 |
|  |  |  |  |  |  |  |  |  |  |
| **Red algae** |  |  |  |  |  |  |  |  |  |
| *Asparagopsis* | 15.5 ± 0.2 | **997 ± 6** | 58657 ± 771 | 14700 ± 127 | **4730 ± 60** | 34.2 ± 0.2 | 1.6 ± 0.03 | 55508 ± 294 | 12800 ± 167 |
| *Halymenia* | 2.0 ± 0.06 | 75.1 ± 0.5 | 48842 ± 1371 | **36600 ± 172** | **9010 ± 19** | 8.3 ± 0.1 | 0.7 ± 0.03 | 21685 ± 388 | 36000 ± 290 |
| *Hypnea* | 5.3 ± 0.08 | **3790 ± 22** | 34898 ± 855 | 19300 ± 246 | **7020 ± 37** | 115 ± 2 | 1.0 ± 0.07 | 14348 ± 159 | 54400 ± 504 |
| *Laurencia* | 4.5 ± 0.06 | **2930 ± 18** | 44524 ± 13 | 12300 ± 100 | **6020 ± 53** | 63.8 ± 0.8 | 1.1 ± 0.08 | 18878 ± 1417 | 64000 ± 1200 |
|  |  |  |  |  |  |  |  |  |  |
| Cottonseed | 11.2 ± 0.2 | 112 ± 4 | 64058 ± 1140 | 15900 ± 109 | **7220 ± 12** | 17.2 ± 1.2 | 1.6 ± 0.06 | 79583 ± 641 | 2080 ± 12 |
| Flinders Grass | 3.4 ± 0.08 | 757 ± 6 | 53420 ± 8 | 7750 ± 148 | 1050 ± 14 | 54.8 ± 0.8 | 1.8 ± 0.1 | 4412 ± 698 | 868 ± 7 |

**Table S2 –** Continuation.

| **Species** | **Ni**^ | **O** | **P** | **Pb*** | **S**^ | **Se**^ | **Sr*** | **V** | **Zn**^ |
| --- | --- | --- | --- | --- | --- | --- | --- | --- | --- |
| **Freshwater green algae** |  |  |  |  |  |  |  |  |  |
| *C vagabunda* | 0.4 ± 0.01 | 353500 ± 141 | 1380 ± 24 | 0.5 ± 0.01 | **11227 ± 812** | 1.07 ± 0.12 | 31.7 ± 0.6 | 0.35 ± 0.01 | 15.5 ± 0.3 |
| *Oedogonium* | 0.8 ± 0.01 | 373300 ± 1273 | 4950 ± 32 | 1.4 ± 0.02 | 2900 ± 420 |  | 17.7 ± 0.3 | 0.60 ± 0.01 | 51.4 ± 0.5 |
| *Spirogyra* | 0.6 ± 0.02 | 412450 ± 1202 | 274 ± 21 | 0.3 ± 0.002 | 3100 ± 170 |  | 132 ± 3 | 0.86 ± 0.02 | 10.9 0.1 |
|  |  |  |  |  |  |  |  |  |  |
| **Marine green algae** |  |  |  |  |  |  |  |  |  |
| *Caulerpa* | 1.7 ± 0.06 | 326650 ± 1909 |  | 0.1 ± 0.003 | **22051 ± 891** | **1.98 ± 0.18** | 67.4 ± 1.8 | 0.91 ± 0.04 | 13.6 ± 0.2 |
| *Chaetomorpha* | 1.5 ± 0.03 | 363600 ± 1131 |  | 0.3 ± 0.003 | **21415 ± 554** |  | 47.1 ± 0.5 | 1.36 ± 0.01 | 64 ± 0.6 |
| *Cladophora* | 2.9 ± 0.05 | 330150 ± 212 | 2320 ± 38 | 0.7 ± 0.008 | **21021 ± 2074** |  | 67.6 ± 1.7 | 4.55 ± 0.06 | 30 ± 0.5 |
| *C. patentiramea* | 4.7 ± 0.04 | 336700 ± 2970 |  | 1.5 ± 0.02 | **32778 ± 839** | **2.51 ± 0.19** | 131 ± 1 | 5.19 ± 0.13 | 19.1 ± 0.4 |
| *Derbesia* | 1.7 ± 0.06 | 312100 ± 1273 | 2340 ± 47 | 1.3 ± 0.01 | **12308 ± 538** | 1.39 ± 0.05 | 31.3 ± 0.6 | 1.17 ± 0.03 | 34.5 ± 0.8 |
| Ulva sp. | 1.9 ± 0.01 | 379000 ± 1131 | 1860 ± 47 | 0.3 ± 0.006 | **28244 ± 827** | 1.25 ± 0.16 | 117 ± 2 | 1.1 ± 0.01 | 25.3 ± 0.3 |
| *U. ohnoi* | 3.0 ± 0.08 | 459350 ± 1768 |  | 0.1 ± 0.003 | **57464 ± 1055** |  | 49.7 ± 1.1 | 0.29 ± 0.01 | 39.6 ± 0.6 |
|  |  |  |  |  |  |  |  |  |  |
| **Brown algae** |  |  |  |  |  |  |  |  |  |
| *Cystoseira* | 1.4 ± 0.05 | 386000 ± 1414 |  | 0.3 ± 0.005 | **13138 ± 837** |  | 1230 ± 27 | 1.89 ± 0.04 | 13.6 ± 0.2 |
| *Dictyota* | 4.5 ± 0.09 | 360350 ± 71 |  | 3.1 ± 0.01 | **11975 ± 247** |  | 1180 ± 10 | 5.47 ± 0.08 | 99.5 ± 1.4 |
| *Hormophysa* | 4.0 ± 0.08 | 394350 ± 1344 |  | 2.8 ± 0.02 | **13375 ± 780** |  | 905 ± 34 | 5.34 ± 0.08 | 56.7 ± 0.5 |
| *Padina* | 2.7 ± 0.06 | 377450 ± 778 |  | 0.5 ± 0.457 | **33734 ± 1514** |  | 1500 ± 25 | 2.05 ± 0.04 | 10.5 ± 0.2 |
| *Sargassum* | 1.8 ± 0.05 | 384800 ± 566 |  | 0.3 ± 0.004 | **9600 ± 1025** | 1.4 ± 0.21 | 1700 ± 27 | 1.72 ± 0.04 | 13.7 ± 0.2 |
| *Colpomenia* | 8.0 ± 0.1 | 324650 ± 2192 |  | 2.4 ± 0.01 | **7200 ± 552** |  | 1500 ± 34 | 9.41 ± 0.29 | 45.3 ± 0.6 |
|  |  |  |  |  |  |  |  |  |  |
| **Red algae** |  |  |  |  |  |  |  |  |  |
| *Asparagopsis* | 1.6 ± 0.03 | 355300 ± 2687 | 70.5 ± 23.5 | 0.4 ± 0.006 | **26871 ± 442** | **38.8 ± 3.7** | 56.5 ± 1.3 | 0.90 ± 0.01 | 145 ± 2 |
| *Halymenia* | 0.7 ± 0.2 | 407550 ± 354 |  |  | **55744 ± 1350** | 1.16 ± 0.15 | 71.7 ± 1 | 0.93 ± 0.01 | 98 ± 1.8 |
| *Hypnea* | 5.1 ± 0.09 | 353500 ± 2687 |  | 1.3 ± 0.02 | **41576 ± 3596** | **4.32 ± 0.26** | 441 ±7 | 10.6 ± 0.3 | 19.1 ± 0.4 |
| *Laurencia* | 4.4 ± 0.05 | 329950 ± 2333 |  | 1.0 ± 0.021 | **27133 ± 735** | **18.9 ± 0.4** | 309 ± 6 | 5.65 ± 0.11 | 23.2 ± 0.3 |
|  |  |  |  |  |  |  |  |  |  |
| Cottonseed | 2.0 ± 0.04 | 331522 ± 1441 | 12700 ± 100 | 0.5 ± 0.007 | 3111 ± 155 |  | 11.2 ± 0.1 |  | 52.9 ± 1.8 |
| Flinders Grass | 0.7 ± 0.01 | 399000 ± 1131 |  | 0.13 ± 0.003 | 1676 ± 183 |  | 47 ± 0.7 | 0.92 ± 0.01 | 36.6 ± 0.2 |
|  |  |  |  |  |  |  |  |  |  |

Parameters were calculated in mg.kg^-1^ DM; (n = 2-5); * elements toxic or not required by beef cattle; ^minerals required by beef cattle; Numbers in bold are very close or above the maximum tolerable concentrations for beef cattle (NRC, 2000);
