# Supplementary material for: Effects of Marine and Freshwater Macroalgae on In Vitro Total Gas and Methane Production
Source: PLoS One. 2014 Jan 22;9(1):e85289. doi: 10.1371/journal.pone.0085289 (PMC3898960; doi:10.1371/journal.pone.0085289)
Supplement: Table S3 — Fatty acid profiles (±SD) of macroalgae species, decorticated cottonseed meal (DCS) and Flinders grass hay. (DOCX) [file pone.0085289.s003.docx]

|  | *C. vagabunda* | *Oedogonium* | *Spirogyra* | *C.aulerpa* | *Chaetomorpha* | *C. coelothrix* | *C. patentiramea* | *Derbesia* | *Ulva* sp. | *U. ohnoi* | *Cystoseira* |
| --- | --- | --- | --- | --- | --- | --- | --- | --- | --- | --- | --- |
| C 12:0 | 0.21 ± 0.04 |  |  |  |  |  |  | 0.44 ± 0.02 |  |  |  |
| C 14:0 | 5.59 ± 0.27 | 0.56 ± 0.04 | 0.33 ± 0.04 | 0.46 ± 0.03 | 1.67 ± 0.04 | 2.34 ± 0.16 | 1.40 ± 0.09 | 1.28 ± 0.06 | 0.32 ± 0.02 | 0.24 ± 0.01 | 0.86 ± 0.02 |
| C 15:0 | 0.51 ± 0.05 | 0.59 ± 0.03 | 0.26 ± 0.04 | 0.29 ± 0.02 | 0.34 ± 0.02 | 0.39 ± 0.05 | 0.27 ± 0.03 | 0.87 ± 0.02 | 0.46 ± 0.03 | 0.33 ± 0.02 | 0.26 ± 0.01 |
| C 16:0 | 8.67 ± 0.65 | 11.46 ± 0.37 | 7.39 ± 0.65 | 7.81 ± 0.16 | 5.08 ± 0.05 | 7.20 ± 0.27 | 5.18 ± 0.25 | 17.29 ± 0.79 | 7.95 ± 0.15 | 5.37 ± 0.01 | 6.19 ± 0.40 |
| C16:1 (7) | 0.94 ± 0.04 | 0.48 ± 0.03 | 0.66 ± 0.06 | 0.33 ± 0.05 | 0.41 ± 0.02 | 0.45 ± 0.03 |  | 0.92 ± 0.01 | 0.94 ± 0.02 | 0.56 ± 0.06 | 0.30 ± 0.02 |
| C 16:1 (9) | 1.08 ± 0.00 | 0.70 ± 0.33 | 0.47 ± 0.00 | 0.87 ± 0.02 | 0.62 ± 0.03 | 1.43 ± 0.03 | 0.57 ± 0.04 | 1.08 ± 0.01 | 0.46 ± 0.05 | 0.73 ± 0.05 | 0.83 ± 0.06 |
| C16:2 (7,10) | 0.67 ± 0.07 | 0.94 ± 0.00 | 0.44 ± 0.05 | 0.69 ± 0.01 | 0.32 ± 0.02 | 0.48 ± 0.02 | 0.29 ± 0.02 | 0.52 ± 0.03 | 0.37 ± 0.03 |  |  |
| C16:2 (9,12) | 3.95 ± 0.48 | 0.47 ± 0.02 |  |  | 1.81 ± 0.04 | 1.20 ± 0.10 | 0.51 ± 0.05 |  |  |  |  |
| C 17:0 |  |  | 0.23 ± 0.03 | 0.23 ± 0.02 |  |  |  | 0.26 ± 0.01 | 0.25 ± 0.03 | 0.22 ± 0.02 | 0.21 ± 0.01 |
| C 17:1 (cis - 10) | 0.32 ± 0.04 |  |  |  |  |  |  | 0.28 ± 0.00 | 0.24 ± 0.01 | 0.26 ± 0.02 |  |
| C16:3 (7, 10, 13) | 0.44 ± 0.05 | 2.75 ± 0.02 | 2.27 ± 0.25 | 2.16 ± 0.12 |  |  |  | 3.64 ± 0.12 | 1.01 ± 0.07 |  |  |
| C16:4 (4,7,10,13) | 0.49 ± 0.08 | 4.99 ± 0.14 |  |  | 1.13 ± 0.05 | 2.03 ± 0.18 | 0.47 ± 0.06 |  | 1.60 ± 0.12 | 0.62 ± 0.01 |  |
| C 18:0 | 0.30 ± 0.03 | 0.61 ± 0.01 | 0.34 ± 0.05 | 0.30 ± 0.02 | 0.23 ± 0.01 | 0.43 ± 0.05 | 0.32 ± 0.04 | 0.61 ± 0.04 | 0.32 ± 0.03 | 0.26 ± 0.02 | 0.32 ± 0.01 |
| C 18:1 ( 9)cis | 7.97 ± 0.60 | 1.74 ± 0.04 | 0.97 ± 0.13 | 0.32 ± 0.03 | 0.76 ± 0.02 | 2.08 ± 0.15 | 1.63 ± 0.04 | 2.13 ± 0.12 | 0.39 ± 0.04 | 0.22 ± 0.04 | 2.24 ± 0.02 |
| C 18:1 (11) | 1.39 ± 0.05 | 0.70 ± 0.02 | 0.35 ± 0.05 | 0.54 ± 0.02 | 0.74 ± 0.02 | 2.36 ± 0.11 | 1.32 ± 0.03 | 1.46 ± 0.03 | 0.97 ± 0.02 | 1.74 ± 0.02 | 0.30 ± 0.02 |
| C 18:2 (9,12) Cis | 6.58 ± 0.43 | 4.23 ± 0.08 | 2.51 ± 0.17 | 1.92 ± 0.02 | 4.85 ± 0.09 | 4.92 ± .024 | 1.50 ± 0.05 | 2.56 ± 0.15 | 1.89 ± 0.09 | 0.39 ± 0.03 | 0.90 ± 0.03 |
| C18:3 ( 6,9,12 ) | 3.42 ± 0.26 | 0.63 ± 0.04 | 0.63 ± 0.00 | 0.46 ± 0.03 | 0.29 ± 0.02 | 0.24 ± 0.00 |  | 0.80 ± 0.00 | 0.29 ± 0.00 | 0.23 ± 0.02 | 0.29 ± 0.03 |
| C 18:3 ( 9,12,15) | 1.26 ± 0.10 | 15.80 ±0.57 | 6.59 ± 0.64 | 4.25 ± 0.02 | 0.67 ± 0.03 | 1.41 ± 0.10 | 0.45 ± 0.02 | 7.96 ± 0.49 | 5.29 ± 0.36 | 1.17 ± 0.02 | 1.04 ± 0.06 |
| C18:4 (6,9,12,15) | 0.30 ± 0.03 | 0.84 ± 0.02 | 0.58 ± 0.06 | 0.55 ± 0.01 | 0.43 ± 0.02 | 0.24 ± 0.02 |  | 0.99 ± 0.01 | 0.84 ± 0.03 | 1.34 ± 0.01 | 0.78 ± 0.04 |
| C 20:0 |  |  |  |  |  | 0.18 ± 0.02 |  | 0.22 ± 0.00 |  |  |  |
| C 20:1 (11) | 0.40 ± 0.05 | 0.57 ± 0.01 |  |  |  | 0.18 ± 0.03 |  | 0.23 ±0.02 |  |  |  |
| C 20:2 (11,14) | 0.31 ± 0.04 | 0.41 ± 0.00 | 0.28 ± 0.04 | 0.24 ± 0.03 | 0.20 ± 0.02 | 0.20 ± 0.03 |  |  |  |  | 0.19 ± 0.02 |
| C 21:0 |  |  |  | 0.21 ± 0.07 | 0.19 ± 0.04 | 0.22 ± 0.09 |  |  |  |  |  |
| C 20:3 (8,11,14) | 0.48 ± 0.06 | 0.26 ± 0.00 | 0.26 ± 0.04 | 0.24 ± 0.02 | 0.21 ± 0.02 | 0.19 ± 0.03 |  | 0.30 ± 0.03 | 0.33 ± 0.04 |  | 0.34 ± 0.00 |
| C 20:4 ( 5,8,11,14) | 2.79 ± 0.28 | 1.14 ± 0.05 | 0.98 ± 0.07 | 0.81 ± 0.04 | 0.52 ± 0.04 | 0.59 ± 0.05 | 0.77 ± 0.05 | 1.42 ± 0.06 | 0.46 ± 0.04 | 0.17 ± 0.02 | 2.52 ± 0.07 |
| C 20:3 (11,14,17) |  | 0.66 ± 0.01 | 0.35 ± 0.01 | 0.22 ± 0.02 |  |  |  |  | 0.19 ± 0.01 | 0.18 ± 0.03 |  |
| C 22:0 | 0.26 ± 0.03 |  | 0.52 ± 0.04 |  |  | 0.21 ± 0.03 |  | 0.94 ± 0.07 | 0.47 ± 0.06 | 0.52 ± 0.03 |  |
| C 20:5 (5,8,11,14,17) | 0.46 ± 0.07 | 2.01 ± 0.00 | 1.12 ± 0.07 | 1.32 ± 0.05 | 0.37 ± 0.03 | 0.75 ± 0.07 | 0.34 ± 0.04 | 0.96 ± 0.06 | 0.34 ± 0.02 | 0.20 ± 0.00 | 0.86 ± 0.06 |
| C 24:0 | 0.79 ± 0.02 |  | 0.33 ± 0.03 | 0.87 ± 0.06 | 0.28 ± 0.02 | 0.40 ± 0.03 | 0.26 ± 0.01 | 1.58 ± 0.07 | 0.24 ± 0.03 |  | 0.26 ± 0.02 |
|  |  |  |  |  |  |  |  |  |  |  |  |
| Total FA | 49.60 ± 3.76 | 57.77 ± 0.70 | 27.88 ± 2.55 | 25.50 ± 0.64 | 21.09 ± 0.45 | 30.83 ± 1.82 | 15.56 ± 0.78 | 48.74 ± 2.00 | 25.63 ± 1.14 | 14.75 ± 0.39 | 18.69 ± 0.72 |
| PUFA | 21.15 ±1.93 | 35.14 ± 0.77 | 16.01 ± 1.41 | 13.27 ± 0.30 | 10.79 ± 0.38 | 12.67 ± 0.94 | 4.34 ± 0.19 | 19.16 ± 0.87 | 12.60 ± 0.81 | 4.30 ± 0.13 | 6.92 ± 0.28 |
| MUFA | 12.11 ± 0.77 | 4.40 ± 0.44 | 2.45 ± 0.24 | 2.27 ± 0.15 | 2.71 ± 0.04 | 7.01 ± 0.27 | 3.53 ± 0.11 | 6.09 ± 0.05 | 3.01 ± 0.01 | 3.51 ± 0.15 | 3.68 ± 0.07 |
| SFA | 16.35 ± 1.06 | 13.22 ± 0.37 | 9.41 ± 0.89 | 10.16 ± 0.27 | 7.78 ± 0.07 | 11.37 ± 0.70 | 7.70 ± 0.49 | 23.49 ± 1.07 | 10.02 ± 0.34 | 6.94 ± 0.11 | 8.10 ± 0.37 |

**Table S3 –** Continuation.

|  | *Dictyota* | *Hormophysa* | *Padina* | *Sargassum* | *Colpomenia* | *Asparagopsis* | *Halymenia* | *Laurencia* | *Hypnea* | DCS | Flinders grass |
| --- | --- | --- | --- | --- | --- | --- | --- | --- | --- | --- | --- |
| C 12:0 |  |  |  |  |  | 0.19 ± 0.00 |  |  |  |  | 0.30 ± 0.02 |
| C 14:0 | 2.29 ± 0.01 | 0.62 ± 0.02 | 0.76 ± 0.02 | 0.70 ± 0.01 | 1.51 ± 0.02 | 1.58 ± 0.04 | 0.25 ± 0.00 | 1.43 ± 0.05 | 0.90 ± 0.06 | 0.27 ± 0.00 | 0.26 ± 0.01 |
| C 15:0 | 0.36 ± 0.01 | 0.25 ± 0.01 | 0.30 ± 0.02 | 0.26 ± 0.01 | 0.32 ± 0.00 | 0.30 ± 0.00 | 0.23 ± 0.00 | 0.27 ± 0.08 | 0.24 ± 0.02 |  | 0.18 ± 0.01 |
| C 16:0 | 7.15 ± 0.16 | 3.40 ± 0.17 | 5.06 ± 0.20 | 3.86 ± 0.11 | 5.34 ± 0.05 | 10.71 ± 0.22 | 6.55 ± 0.11 | 5.16 ± 0.30 | 4.19 ± 0.02 | 6.64 ± 0.03 | 1.14 ± 0.13 |
| C16:1 (7) | 0.31 ± 0.00 | 0.21 ± 0.02 | 0.26 ± 0.00 | 0.30 ± 0.01 | 0.36 ± 0.05 | 0.22 ± 0.01 | 0.30 ± 0.00 |  | 0.22 ± 0.03 |  | 0.19 ± 0.01 |
| C 16:1 (9) | 0.43 ± 0.01 | 0.52 ± 0.02 | 0.76 ± 0.03 | 0.69 ± 0.04 | 0.49 ± 0.02 | 0.51 ± 0.01 | 0.42 ± 0.05 | 0.56 ± 0.08 | 0.77 ± 0.03 | 0.31 ± 0.02 |  |
| C16:2 (7,10) |  |  |  |  |  | 0.16 ± 0.00 |  |  |  |  |  |
| C16:2 (9,12) |  |  |  |  |  |  |  |  |  |  | 0.20 ± 0.01 |
| C 17:0 |  | 0.24 ± 0.01 | 0.22 ± 0.02 | 0.21 ± 0.01 | 0.20 ± 0.00 | 0.22 ± 0.00 |  |  | 0.21 ± 0.03 | 0.19 ± 0.00 |  |
| C 17:1 (cis - 10) |  |  |  |  |  |  |  |  |  |  |  |
| C16:3 (7, 10, 13) |  |  |  |  |  | 0.27 ± 0.00 |  |  |  |  |  |
| C16:4 (4,7,10,13) |  | 0.17 ± 0.01 |  |  |  | 0.17 ± 0.00 |  |  | 0.21 ± 0.03 |  |  |
| C 18:0 | 0.65 ± 0.00 | 0.28 ± 0.01 | 0.40 ± 0.03 | 0.27 ± 0.01 | 0.33 ± 0.01 | 0.38 ± 0.00 | 0.23 ± 0.09 | 0.32 ± 0.01 | 0.28 ± 0.03 | 1.00 ± 0.00 | 0.36 ± 0.03 |
| C 18:1 ( 9)cis | 5.07 ± 0.04 | 1.55 ± 0.03 | 2.28 ± 0.03 | 1.39 ± 0.01 | 3.03 ± 0.02 | 1.38 ± 0.05 | 1.45 ± 0.03 | 1.44 ± 0.05 | 1.00 ± 0.06 | 4.63 ± 0.01 | 0.59 ± 0.12 |
| C 18:1 (11) | 0.44 ± 0.01 | 0.32 ± 0.00 | 0.37 ± 0.02 | 0.29 ± 0.02 | 0.49 ± 0.02 | 0.80 ± 0.00 | 0.61 ± 0.00 | 0.51 ± 0.03 | 0.39 ± 0.01 |  | 0.20 ± 0.02 |
| C 18:2 (9,12) Cis | 0.74 ± 0.02 | 2.16 ± 0.07 | 0.75 ± 0.02 | 0.66 ± 0.01 | 0.53 ± 0.01 | 0.49 ± 0.02 | 0.27 ± 0.00 | 0.42 ± 0.01 | 0.30 ± 0.04 | 12.98 ± 0.15 | 1.07 ± 021 |
| C18:3 ( 6,9,12 ) | 0.36 ± 0.00 | 0.44 ± 0.00 | 0.36 ± 0.01 | 0.32 ± 0.03 | 0.30 ± 0.00 | 0.35 ± 0.01 | 0.26 ± 0.03 | 0.29 ± 0.05 | 0.26 ± 0.04 |  |  |
| C 18:3 ( 9,12,15) | 1.16 ± 0.01 | 0.67 ± 0.01 | 1.14 ± 0.01 | 0.81 ± 0.02 | 0.43 ± 0.01 | 0.71 ± 0.02 |  | 0.23 ± 0.00 | 0.22 ± 0.04 | 0.23 ± 0.00 | 0.57 ± 0.03 |
| C18:4 (6,9,12,15) | 3.40 ± 0.03 | 0.65 ± 0.02 | 2.30 ± 0.06 | 0.88 ± 0.02 | 0.72 ± 0.04 | 0.98 ± 0.02 |  | 0.31 ± 0.04 | 0.32 ± 0.02 |  |  |
| C 20:0 | 0.37 ± 0.02 |  | 0.25 ± 0.01 |  | 0.32 ± 0.00 |  |  |  |  | 0.25 ± 0.00 | 0.49 ± 0.06 |
| C 20:1 (11) |  |  |  |  |  | 0.23 ± 0.01 |  |  | 0.19 ± 0.03 |  |  |
| C 20:2 (11,14) |  | 0.39 ± 0.00 |  | 0.21 ± 0.01 |  | 0.26 ± 0.01 |  |  |  |  |  |
| C 21:0 |  |  |  |  |  | 0.16 ± 0.00 |  |  |  |  | 0.19 ± 0.01 |
| C 20:3 (8,11,14) | 0.36 ± 0.00 | 2.45 ± 0.16 | 0.47 ± 0.01 | 0.31 ± 0.02 | 0.24 ± 0.01 | 0.25 ± 0.00 | 0.20 ± 0.01 | 0.29 ± 0.02 | 0.22 ± 0.03 |  |  |
| C 20:4 ( 5,8,11,14) | 2.21 ± 0.04 | 3.65 ± 0.11 | 2.08 ± 0.03 | 1.74 ± 0.01 | 1.42 ± 0.03 | 3.83 ± 0.58 | 1.16 ± 0.07 | 1.58 ± 0.07 | 0.72 ± 0.05 |  |  |
| C 20:3 (11,14,17) |  |  |  |  |  |  |  |  |  |  |  |
| C 22:0 |  |  |  |  | 0.21 ± 0.01 |  |  |  |  |  | 0.38 ± 0.03 |
| C 20:5 (5,8,11,14,17) | 1.69 ± 0.03 | 0.57 ± 0.12 | 0.64 ± 0.03 | 0.74 ± 0.02 | 1.23 ± 0.01 | 2.65 ± 0.38 | 1.03 ± 0.04 | 3.25 ± 0.18 | 1.09 ± 0.04 |  |  |
| C 24:0 |  | 0.21 ± 0.00 |  | 0.29 ± 0.06 | 0.30 ± 0.00 | 0.22 ± 0.02 |  |  | 0.26 ± 0.02 |  | 0.49 ± 0.03 |
|  |  |  |  |  |  |  |  |  |  |  |  |
| Total FA | 27.01 ± 0.12 | 18.77 ± 0.18 | 18.39 ± 0.17 | 13.93 ± 0.20 | 18.30 ± 0.34 | 27.28 ± 1.32 | 12.97 ± 0.18 | 16.06 ± 0.34 | 11.99 ± 0.51 | 26.51 ± 0.22 | 6.62 ± 0.66 |
| PUFA | 9.93 ± 0.04 | 11.15 ± 0.25 | 7.73 ± 0.04 | 5.67 ± 0.13 | 4.86 ± 0.12 | 10.13 ± 1.04 | 2.92 ± 0.15 | 6.37 ± 0.14 | 3.34 ± 0.28 | 13.21 ± 0.15 | 1.84 ± 0.19 |
| MUFA | 6.24 ± 0.04 | 2.61 ± 0.04 | 3.67 ± 0.02 | 2.67 ± 0.07 | 4.90 ± 0.13 | 3.53 ± 0.04 | 2.78 ± 0.01 | 2.51 ± 0.06 | 2.58 ± 0.10 | 4.95 ± 0.02 | 1.18 ± 0.15 |
| SFA | 10.83 ± 0.11 | 5.01 ± 0.11 | 6.99 ± 0.11 | 5.58 ± 0.00 | 8.53 ± 0.10 | 13.77 ± 0.25 | 7.27 ± 0.02 | 7.18 ± 0.26 | 6.07 ± 0.13 | 8.35 ± 0.04 | 3.80 ± 0.32 |

Parameters were calculated in mg.g^-1^ DM; (n = 2); Total FA, total fatty acids; PUFA, polyunsaturated fatty acids; MUFA, monounsaturated fatty acids; SFA; saturated fatty acids.
